# The Biotechnological Potential of *Pediococcus* spp. Isolated from Kombucha Microbial Consortium

**DOI:** 10.3390/foods9121780

**Published:** 2020-12-01

**Authors:** Camelia Filofteia Diguță, George Daniel Nițoi, Florentina Matei, Gabriela Luță, Călina Petruța Cornea

**Affiliations:** Faculty of Biotechnologies, University of Agronomic Sciences and Veterinary Medicine Bucharest, 59 Mărăști Blvd., 011464 Bucharest, Romania; camifilo@yahoo.com (C.F.D.); daniel.nitoi0@gmail.com (G.D.N.); glutza@yahoo.com (G.L.); pccornea@yahoo.com (C.P.C.)

**Keywords:** lactic acid bacteria (LAB), *Pediococcus* spp., Kombucha, probiotic, antimicrobial, lyophilization

## Abstract

In the past decade, the probiotic market has grown rapidly, both for foods and supplements intended to enhance wellness in healthy individuals. Different lactic acid bacteria (LAB), especially *Lactobacillus* spp., of different origins have already been used to develop commercial probiotic products. Nowadays, LAB new alternative sources, such as non-dairy fermented food products, are being exploited. One such source is Kombucha, a fermented low-alcohol beverage made of tea leaves. In this regard, we tested seven *Pediococcus* spp. strains isolated from a local industrial Kombucha for their biotechnological potential. Two, out of the seven isolates, identified as *Pediococcus pentosaceus* (L3) and *Pediococcus acidiliactici* (L5), were selected as successful candidates for the food industry, due to their probiotic and technological properties. In regard to their resistance in the gastro-intestinal tract, both selected strains were tolerant to a pH of 3.5, presence of 0.3% pepsin, and 0.5% bile salt concentration. On the antagonistic side, the fresh suspension of selected isolates had high inhibitory activity against pathogenic bacteria, such as *Salmonella enterica* Typhimurium, *Listeria monocytogenes, Listeria ivanovii, Bacillus cereus, Proteus hauseri,* and methicillin resistant *Staphylococcus aureus*. In addition, moderate to high inhibitory activity was noticed against foodborne molds (e.g., *Penicillium expansum* and *Penicillium digitatum*). These safety issues were supported by their negative hemolytic activity and good antioxidant potential (56–58%). Selected isolates were sensitive to ampicillin, penicillin, erythromycin, and lincomycin, while a broad range of other antibiotics were not effective inhibitors. On the technological side, both strains tolerated 5% NaCl and, during the freeze-drying process, had a good survival rate (86–92%). The selected *Pediococcus* strains have proven properties to be used for further development of functional products.

## 1. Introduction

In 2002, the United Nations’ Food and Agriculture Organization and the World Health Organization [1] defined probiotics as “live microorganisms that, when administered in adequate amounts, confer a health benefit on the host”. From that moment until the present, important efforts have been made to develop probiotic commercial products, as well as products with prebiotics and synbiotics. It is generally accepted that these are likely to provide general health benefits for humans and animals, such as restoring the disturbed gut microbiota, regulating intestinal transit, competitively excluding pathogens from adhesion sites, and producing short chain fatty acids [2]. Consequently, the probiotic market has grown rapidly both for foods and supplements intended to enhance wellness in healthy individuals [3]. Different microorganisms of different origins have already been used to develop commercial probiotic products. The most common commercially available strains belong to the *Lactobacillus* species (*casei, acidophilus, fermentum, gasseri, johnsonii, reuteri, plantarum, paracasei, rhamnosus* or *salivarius*) and *Bifidobacterium* species (*bifidum, breve, adolescentis, animalis,* or *longum*) [4]. The probiotic market trend indicates that there is still room for new products made of alternative probiotic sources that bode extended shelf lives, chemical stability, and are reasonably priced [5], all while reducing the risk of cholesterol problems in lactose intolerant people [6]. Different alternative sources have been exploited, i.e., mainly non-dairy fermented food products, such as traditional fermented foods, traditional fermented drinks, vegetables, and fruit juice. It has been demonstrated that the differences in raw materials and ingredients used to prepare such products are the main factors that lead to the different available species or strains of probiotics in food sources [7].

The main lactic acid bacteria (LAB) genera isolated from fermented plants and fermented meats is *Lactobacillus*. The most common LAB genera isolated from fermented seafoods is *Enterococcus* [7]. For example, the main LAB isolated from black olives were *L. pentosus* and *Leuconostoc mesenteroides* and from green olives were *L. pentosus*, *L. plantarum*, and *L. paracasei* [8,9]. *Pediococcus pentosaceous* strains were isolated from some traditional Thai fermented foods containing fish and pork [10] or from traditional Ethiopian fermented beef sausage [11].

Non-dairy fermented beverages, such as *Pozol*, *Bushera*, *Boza*, *Mahewu*, and *Togwa,* made of cereals, millets, legumes, fruits, and vegetables, are frequently reported as good sources of probiotics [12]. In the past decade, special attention has been paid to Kombucha, a fermented low-alcohol beverage made of tea leaves. Kombucha is reported to have several health benefits, such as antioxidant potential, antibacterial activity, and antiproliferative activity against cancer cell lines [13]. Different sources of Kombucha were characterized for their biochemical composition and complex microbial biodiversity (characterized by the presence of acetic acid bacteria, yeasts, and lactic acid bacteria). Generally, the consortium is dominated by acetic bacteria such as *Komagataeibacter* sp. and *Gluconobacter* sp., as well as yeast such as *Brettanomyces, Hanseniaspora*, *Saccharomyces, Torulaspora,* or *Schizosaccharomyces* [14,15]. Few studies reported on the interactions inside such complex microbial biodiversity and the focus was mainly on the acetic bacteria-yeast interaction [16]. The LAB are often, but not always, reported in Kombucha, and the main identified genera are *Lactobacillus* spp., *Lactococcus* spp., or *Leuconostoc* spp. [17,18]. Occasionally, *Pediococcus* spp. has been reported to belong to such consortium in industrial Kombucha sources [19]. It has been demonstrated that the viability of probiotic microorganisms is more difficult to maintain in non-dairy matrices than in dairy matrices and the physicochemical parameters must be carefully controlled to guarantee the probiotic viability [20]. Different strains of *Pediococcus* spp. isolated from other traditional fermented sources were reported as potential probiotics: *P. pentosaceus* from *Idly* batter, a traditional fermented food in South India [21]; *P. pentosaceus* and *P. acidiliactici* from *Omegisool*, a traditionally fermented millet alcoholic beverage in Korea [22]; *P. pentosaceous* strains isolated from *Wakalim*, the traditional Ethiopian fermented beef sausage [11]; or from *Kunu-zaki*, a Nigerian traditional fermented drink [23].

In the developmental process of new probiotic food products, the selection of probiotic microorganisms is the main challenge for food industries. The selection of probiotics from different sources requests screening for non-pathogenic microorganisms, which are further evaluated for some basic properties, such as the tolerance to gastro-intestinal environments, the ability to inhibit pathogens in the gastro-intestinal tract, resistance to antibiotics, adhesion potential, etc. Another important feature for industrial use is the viability during processing treatments and storage [24]. Our demarche targeted the screening of *Pediococcus* strains, formerly isolated from industrial Kombucha, for their probiotic potential and technological aspects. On the probiotic side, the isolates were tested for their resistance to the gastro-intestinal environment (i.e., low pH, pepsin, and bile salt presence), antagonism against different groups of pathogens, safety aspects (e.g., hemolytic activity, antioxidant activity), and antibiotic resistance. The technological aspects covered tolerance to NaCl presence, as a food additive, and the influence of the freeze-drying procedure on the cell viability, as a conditioning method for probiotics’ industrial use.

## 2. Materials and Methods

### 2.1. Microorganisms

The tested LAB were formerly isolated in our laboratory from an industrial Kombucha source, made of green tea leaves, and provided by Medica FarmImpex SRL, Otopeni, Romania. All isolates (S1, S2, S3, L3, and F1) have been previously identified by sequencing and reported as belonging to the species *P. pentosaceus* [19], except two isolates (L5 and F2), which reclassified after further investigations as *P. acidilactici* (for F1 and F2 isolates the sequences analysis is provided in the Supplementary Information, Table S1).

In the case of the antagonistic interactions’ study, different groups of pathogens were taken into account, from human pathogenic bacteria to foodborne molds, as detailed in Table 1.

### 2.2. Testing LAB Tolerance to Pepsin in Acidic pH

The tolerance to pepsin’s presence and acidic pH was tested with an adapted method described by Chen et al. 2018 [25]. Fresh culture of LAB cultivated in a Man–Rogosa–Sharpe (MRS) broth medium (VWR, UK) for 18 h at 37 °C was centrifuged at 2000 g/10 min and the cell pellets was re-suspended and washed twice, under aseptic conditions, with sterile physiological saline solution (NaCl 0.9%). The LAB biomass was re-suspended in PBS (phosphate-buffered saline) supplemented with 0.3% pepsin (Merck); the pH was adjusted to 2.5 with 1 N HCl. The suspension was incubated at 37 °C and cell viability was calculated according to the formula:

$$\%\;viability=\;\frac{logCFU\;Nt}{logCFU\;Ni}\;\times \;100$$

where *Ni* is the initial number of viable cells in the suspension and *Nt* the total viable cells after a specific incubation time. Colonies of the surviving LAB were counted after on-plate cultivation at 37 °C during 24 h, on MRS medium (VWR, UK). Samples were counted after 90 and 180 minutes’ exposure to low pH and pepsin.

### 2.3. Testing LAB Tolerance to Bile Salts Presence

Bile salts influence on LAB was tested according to the method described by Adetoye et al. (2018) [26] with some modifications. The Kombucha LAB isolates were cultivated in MRS broth (VWR, UK) at 37 °C/4 h supplemented with different bile salt (Oxoid, UK) concentrations (0.5 and 1%). The sampling was done after 2 and 4 h of cultivation; dilutions were performed in sterile peptone (Oxoid, UK) water and spread on MRS-agar plate. Cell levels dynamic was measured by calculating CFU/mL After counting the colonies by the aid of a Colony Counter (Boeco CC-1, UK). All samples were performed in triplicate.

### 2.4. Testing the Inhibitory Activity of the LAB on Pathogenic Microorganisms by Disk-Diffusion Assay

The antagonistic activity of the Kombucha LAB on the pathogenic microorganisms has been tested by an adapted method of the disk-diffusion assay (Kirby–Bauer test). A double layer media was used; the bottom layer was MRS with 2% agar, while the upper layer contained 1% agar. For the pathogenic bacteria the employed medium was nutrient-agar, while for the yeast and molds PDA (potato dextrose agar) (VWR, UK). Fresh suspension (10 µL) of LAB cultivated on MRS broth for 18 h at 37 °C was inoculated in small wholes made in the first layer medium. After 48 h of LAB incubation at 35 °C, the pathogenic microorganisms were incorporated in the second layer of the media, containing 1% agar. After other 48 h of incubation at 35 °C, the inhibition zones were measured and all plates were performed in triplicate.

### 2.5. Testing the LAB Hemolytic Activity

The hemolytic activity of the LAB isolates was determined using the procedure described by Yadav et al. (2016) [27]. All the isolates tested were streaked onto blood agar plates (Oxoid, UK) containing 5% (*w*/*v*) sheep blood and incubated at 37 °C for 48 h. After incubation, the plates were examined for the zone of hemolysis around bacterial growth. If the zone around bacterial growth was clear, the bacteria were susceptible of β-hemolysis; when the zone become greenish, the tested bacteria produced α-hemolysis; if the microorganism did not produce hemolysins and did not break down the blood cells, no clearing occurred.

### 2.6. Antioxidant Assay

The free radical scavenging activities of the LAB suspension (10^9^ CFU/mL) was determined using the stable free radical diphenyl picryl hydrazyl (DPPH) method according to the procedure adapted by Brand-Williams et al. (1995) [28] for complexes matrices. Briefly, 2 mL of 100 μM methanolic solution of DPPH was mixed with 1 mL of different LAB suspensions. The mixture was shaken vigorously and incubated for 30 min at room temperature, in a dark place. The change in color from deep violet to light yellow was then measured at 515 nm (A_sample_). The percentage of the radical scavenging activity (RSA) was calculated by using the following equation: % RSA = (1− [A_sample_/A_control t=0_])/100. DPPH 100 μM solution in 80% methanol was used as a negative control.

### 2.7. Antibiotic Susceptibility Test

From the seven Kombucha LAB isolates, due to their biotechnological potential, two of them (L3 and L5) were used further to be tested for their antibiotic susceptibility different antibiotics (BioAnalyse, Turkey). The susceptibility of the LAB isolates was assessed on MRS agar plates using the antibiotic disc diffusion method. The overnight LAB cultures (100 μL) were spread on MRS agar plates and allowed to dry. The antibiotic discs were placed on the inoculated plates and incubated at 37 °C for 48 h. In the test we used ampicillin (AM-10), penicillin (P-2U), cephalexin (CL-30), cefaloridine (CPH-30), cefuroxime (sodium) (CXM-30), ceftriaxone (CRO-30), norfloxacin (NOR-30), nalidixic acid (NA-30), amikacin (AK-10), erythromycin (E-10), gentamicin (CN-30), lincomicina (L-10), streptomycin (S-10), chloramphenicol (C-30), trimethoprim/sulphamethoxazole (SXT-1,25/23,75, SXT-25), nitrofurantoin (F-300), colistin (CT-10), vancomycin (VA-10), bacitracin (B-10U), amoxicillin/clavulanic acid, (AMC-20/10, AMC-30), tetracycline (TE-30), kanamycin (K-30), oxitetracycline (T-30), and fluconazole (FLU-10). The diameter of the zone of inhibition was measured using the antibiotic zone scale (CLSI scale). The results obtained are presented in terms of susceptibility, moderate susceptibility, or resistance. These results were compared with the interpretative zone diameters as described in the Performance Standards for Antimicrobial Disc Susceptibility Tests [29].

### 2.8. Testing the Kombucha Selected LAB for NaCl and pH Tolerance

The selected LAB (L3 and L5) were tested for their tolerance to different NaCl concentration and different pH, according to the method described by Prabhurajeshwar and Chandrakanth, (2019) [30] with some modifications. All tests were conducted in MRS broth. The NaCl (VWR, UK) concentrations was adjusted to 2.5%, 5%, 7.5%, 9.5%, and 11.5%; the pH tested values were 3.5, 5.5, 7.5, and 9.5. After the inoculation with 10^6^ CFU/mL, the cultures were incubated at 37 °C for 72 h. The bacterial population evolution was performed by measuring the optical density at 600 nm and by counting the CFU/mL on plates with MRS agar. All experiments were performed in triplicates and ±SD was taken into account.

### 2.9. Testing the Behavior of Kombucha Selected LAB to the Lyophilization Procedure

Both selected isolates, L3 and L5, were subject to the lyophilization procedure. Two different cryoprotectors were used: sterile solution of 5% glucose and 5% sucrose (Oxoid, UK). As a control, sterile distilled water was employed. The lyophilization procedure included the following steps: bacterial biomass obtained after cultivation in MRS broth (37 °C/48 h) was recovered and washed twice with sterile physiological saline solution (NaCl 0.9%); after centrifugation at 5000 rpm/5 min/4 °C, the sediment was transferred in the cryoprotector solution and freezed overnight at −24 °C. As equipment was used a 6 L benchtop freeze-dryer from Labconco, USA. The lyophilization parameters were 0.04 mbar pressure and −55 °C temperature for 6 h. The cell viability was tested before and after the lyophilization procedure. Supplementary, the lyophilized biomass was tested for its antagonistic activity against several pathogenic microorganisms, as described above (Section 2.4).

### 2.10. Statistical Analysis

All of the parameters investigated were evaluated in a minimum of three independent determinations, and the results were expressed as the mean ± standard deviation (SD). The differences were analyzed by one-way analysis of variance (ANOVA). The significance level for the calculations was set as follows: significant, *p ≤* 0.05; very significant, *p ≤* 0.01; and highly significant, *p ≤* 0.001.

## 3. Results

### 3.1. LAB Tolerance to Pepsin Presence in Acid pH

**Figure 1:**
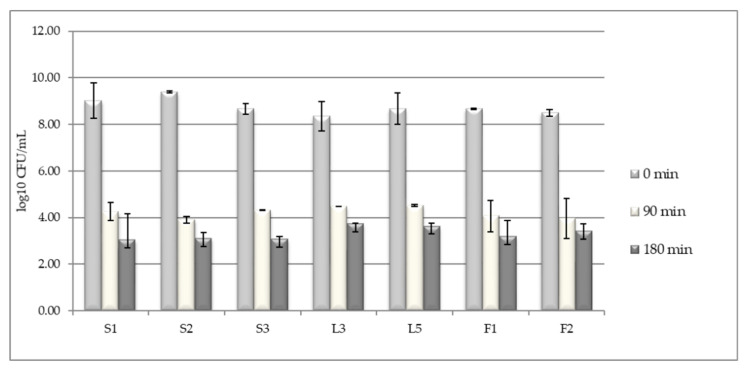
Tolerance of Kombucha lactic acid bacteria (LAB) isolates when cultivating at pH 2.5 and 0.3% pepsin. Data shown are mean ± standard deviation (SD) of triplicate values of independent experiments.

In the intestinal tract, the potential probiotic microorganisms, should tolerate the environmental conditions found in the superior part of the tract, where the pH is very acidic and the pepsin is present. In our study, conducted on a pH of 2.5 and in the presence of 0.3% pepsin, it was noticed that, after 90 min exposure, the viable cell levels substantially decreased from 10^8^–10^9^ CFU/mL to 10^4^ CFU/mL. After a longer exposure, of 90 cultivation minutes (a total of 3 h), the population level decreased with other one logarithmic unit, to 10^3^ CFU/mL (Figure 1).) Isolates like L3, L5, and F2 have better adapted to a very acidic medium than the other isolates.

### 3.2. Testing LAB Tolerance to Bile Salts Presence

**Figure 2:**
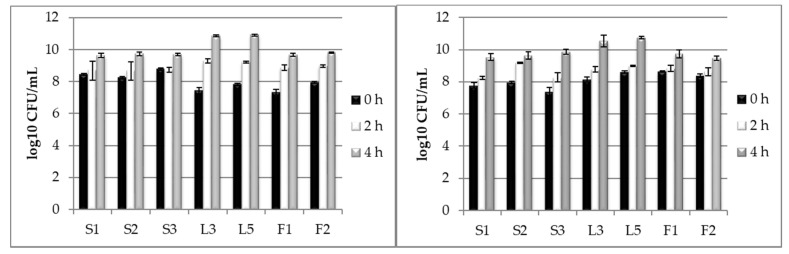
Tolerance of Kombucha LAB isolates to different bile salts concentration: (**a**) −0.5%; (**b**) −1%.

When taking into account the study of microorganisms, LAB has probiotic potential and thus different characteristics should be taken in account, as described above. The bile salt tolerance was tested at concentrations of 0.5% and 1%, while bile salts vary between 0.3% and 0.5% in the human digestive tract. The initial cell levels were 10^7^–10^8^ CFU/mL in all samples; in the first two hours of incubation, the cell levels increased significantly (*p ≤* 0.05) with one logarithmic unit (10^8^–10^9^ CFU/mL) when cultivating under 0.5% bile salts. At 1% salt concentration, no significant increases were measured (Figure 2). Under both concentrations, the isolates L3 and L5 proved to be tolerant to the bile salts presence, with a population level increased (*p ≤* 0.01) to 10^10^ CFU/mL after 4 cultivation hours.

### 3.3. Inhibitory Activity of Kombucha LAB on Pathogenic Microorganisms

All lab isolates were tested for their inhibitory activity against microorganisms with pathogenic potentials. It was noticed that all isolates had high inhibitory activity against the major foodborne pathogens (Table 2) such as *Salmonella enterica* Typhimurium, *Listeria monocytogenes, Listeria ivanovii, Bacillus cereus, Proteus hauseri,* and other human pathogens responsible for major illnesses, such as methicillin resistant or methicillin sensible *Staphylococcus aureus*. The lower inhibitory activity was registered in the case of *E. coli*. Low to moderate inhibitory activity was noticed against the main potential pathogenic *Candida* spp., including a serotype A *Candida albicans*; the isolates S2, L3, and L5 are potential candidates for *C. albicans* moderate inhibition (Figure S1). No inhibitory activity was noticed against *Candida krusei*.

In relation to the foodborne molds, low to medium inhibition was noticed in the case of Aspergilli group. The highest inhibitory activity was exhibited by L3, L5, and F1 isolates on *Aspergillus carbonarius* (Figure S2). On both *Penicillium expansum* and *Penicillium digitatum,* the mycelial growth was inhibited moderately to high by the LAB isolates, except for the S3 isolate.

### 3.4. Hemolytic and Antioxidant Activity of Kombucha LAB Strains

The safety evaluation of the isolates was primarily determined by detecting their hemolytic activity, which proved the nonpathogenic status of the probiotic isolates. The results revealed no hemolytic activity (Figure S3), which was confirmed by the “no zone” in the test plates inoculated with all the isolates studied.

**Figure 3:**
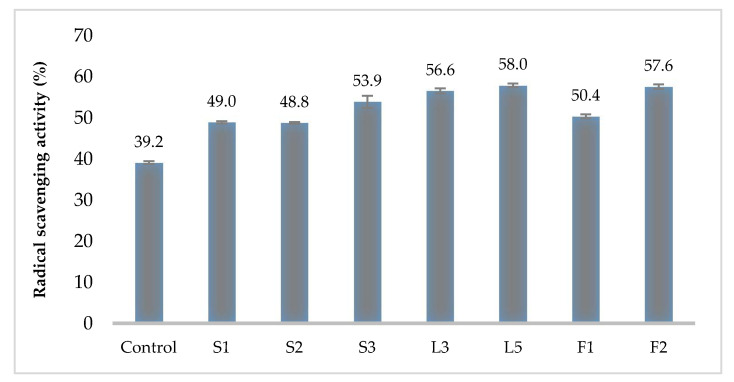
Radical scavenging activity of LAB strains isolated from industrial Kombucha. Data shown are mean ± SD of triplicate values of independent experiments.

Regarding antioxidant activity, the control (MRS media without any inoculum) reached a 39.2% radical scavenging activity. The Kombucha LAB isolates radical scavenging activity varied between 48.8% and 58.0%. Significant differences (*p ≤* 0.05) were noticed between Control and all LAB tested isolates. No significant differences were observed between the isolates L5 (58%), F2 (57.6%), and L3 (56.6%), which showed the highest antioxidant activity (Figure 3).

### 3.5. Antibiotic Susceptibility of Selected LAB Isolates

The antibiotic susceptibility of the selected LAB isolates L3 and L5 was assessed on MRS agar plates using the antibiotic disc diffusion method using abroad range of antibiotics’ classes. The variability of the antibiotic susceptibility can be observed in Table 3. The isolates have relatively similar patterns in their antibiotic susceptibility, with few exceptions, in which L3 isolate showed higher resistance. For both isolates, an important range from the tested antibiotics were not effective inhibitors, including fluoroquinolones, amoxicillin/clavulanic acid, cephalexin, cefuroxime, amikacin, streptomycin, kanamycin, sulphamethoxazole, vancomycin, tetracycline, colistin, and fluconazole. Kombucha’s selected LAB were sensitive to ampicillin, penicillin, erythromycin, and lincomycin.

### 3.6. Tolerance of the Selected LAB to NaCl and pH

**Figure 4:**
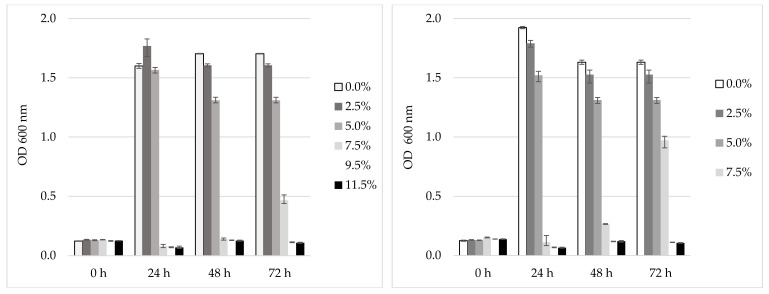
Tolerance to different NaCl concentrations of Kombucha L3 (**left**) and L5 (**right**) LAB isolates. Data shown are mean ± SD of triplicate values of independent experiments.

Both L3 and L5 were tested for their behavior under different NaCl concentrations. The bacterial population dynamic of L3 under such environmental condition is represented in Figure 4. It was noticed that both L3 and L5 tolerate concentrations of 2.5% and 5% NaCl during 72 h of cultivation (*p ≤* 0.05); at the 7.5% NaCl concentration, a slight increase of the population in the case of L3 can be observed, while the increase for L5 was consistent (double that of L3). The level of the viable cells after 72 cultivation hours at 37 °C reached a significant level (*p ≤* 0.05) of 10^12^ CFU/mL in the control and at 2.5% NaCl, at 5% the level was 10^1o^ CFU/mL, for both L3 and L5. At 7.5% NaCl, the maximum population level was 10^7^ CFU/mL, while for L5 it was 10^9^ CFU/mL (data not shown). Regarding the pH tolerance, at pH 3.5 the isolate L5 behaved better (higher population level) than L3, while at higher pH (5, 7.5 and 9.5) both isolates exhibited similar behaviors. The significantly (*p ≤* 0.05) higher population levels (10^12^ CFU/mL) were counted for both isolates in the case of the pH of 7.5.

### 3.7. The LAB Behavior under Lyophilization Procedure

The isolates L3 and L5 were lyophilized in the presence of two different cryoprotectors (glucose and sucrose). The initial viable biomass for both isolates was adjusted to an initial content of 10^10^ CFU/mL. After the lyophilization, in the control (sterile distilled water) the viable cells levels was 10^5^ CFU/mL (a 50% survival), whereas in both sugar solutions, L3 survival was 85–86% and L5 was slightly higher (91–92%). The lyophilized biomass was rehydrated and tested for the inhibitory activity against several pathogens. The severe environmental conditions applied during lyophilization did not affect the inhibitory activity of the rehydrated bacterial biomass on the tested pathogenic microorganisms, as provided in Table 4 (*Listeria* ssp., *Salmonella enterica Thyphimurium, Stahylococcus aureus* methicillin sensible and methicillin-resistant, *Bacillus cereus,* and *Candida albicans).*

## 4. Discussion

Nowadays, alternative isolation sources of probiotics, such as non-dairy fermented food products, are increasingly exploited. Kombucha, a fermented beverage made of tea leaves (*Camellia sinensis*) is one such source because of its complex SCOBY (symbiotic consortia of bacteria and yeast). The main reported LAB in this SCOBY are *Lactobacillus* spp., *Lactococcus* spp., or *Lecunosctoc* spp., while *Pediococcus* spp. was only occasionally isolated [17,18,19]. Our goal was to test the biotechnological potential of seven *Pediococcus* spp. strains isolated from a local industrial Kombucha, and identified formerly as *Pediococcus pentosaceus* [19] and *Pediococcus acidiliactici*. In an initial stage, all isolates were screened for a series of probiotics properties, such as their resistance in the gastro-intestinal tract, antagonistic activity against human/animal pathogens, foodborne molds, antioxidant potential, and hemolytic activity. Two isolates, L3 (*Pediococcus pentosaceus*) and L5 (*Pediococcus acidilactici*), were selected as potential probiotic candidates and further investigated for their antibiotic resistance and for their behavior against some technological parameters. In terms of technological aspects, the strains were tested for their tolerance to a food preservative (sodium chloride) and for their viability after applying extreme freeze-dried conditions, which is a frequent method used industrially to process probiotics. Former data [31] showed that L5 isolate have a good capacity to adhere in vitro to the surface of the Caco-2 cellular monolayer; it was proven that after four incubation hours, the bacterial cells start to form aggregates, suggesting a diffuse-aggregative adherence pattern.

The resistance in the gastro-intestinal tract in the presence of bile salts, pepsin, and acidic pH is one of the most important characteristics of the probiotic microorganisms. *P. pentosaceus* strains isolated from *Wakalim*, a traditional Ethiopian fermented beef sausage, are reported as tolerant to a pH of 3 and a 0.3% bile salt concentration [9], while strains isolated from *Kunu-zaki*, a Nigerian traditional fermented drink made from non-germinated sorghum and millet cereal grains proved to resist at the same pH but to a higher bile salt concentration of 3% [19]. In our case, the selected strains (L3 and L5) were tolerant to a pH of 2.5, 0.3% pepsin and 0.5% bile salt concentrations.

Numerous strains isolated from fermented beverages were reported as having antagonistic effect on pathogenic microorganism, mainly due to the production of organic acids and hydrogen peroxide. LAB strains of *Lactobacillus plantarum* isolated from a Turkish traditional fermented drink (*Boza*) showed antagonistic activity against pathogenic bacteria such as *Listeria monocytogenes, Bacillus subtilis, Bacillus cereus, Yersinia enterocolitica, Pseudomonas aeruginosa, E. coli, Salmonella enterica* Typhimurium, and *Klebsiella pneumonia*, even after the neutralization of the cell free supernatant. The authors imply the presence of bacteriocin’s production [32]. *Kunu-zaki*, the Nigerian traditional fermented beverage, had isolated probiotic strains of *Lactobacillus, Pediococcus,* and *Lactococcus,* all of which were reported with inhibitory activity on *Pseudomonas aeruginosa, Staphylococcus aureus*, *E. coli*, and *Enterococcus faecalis* when used for active cell suspension [23]. The authors suggested that the results were caused by bacteriocins and organic acids production. *P. pentosaceus* isolated from other natural fermented sources, such as Kimchi (traditional Korean fermented vegetable), has been reported to inhibit *Listeria monocytogenes* due to the production of class II bacteriocins [33]. The species of *Pediococcus* genera are recognized for their ability to produce bacteriocin. Oher strains of *P. pentosaceus* isolated from paocai (a Chinese fermented vegetable) have been proven to have inhibitory activity against *E. coli* and *Salmonella enterica* Typhimurium [34]. It has also proven that *P. pentosaceus* strains isolated from traditional Thai meat fermented foods inhibited the growth of some pathogenic bacteria, such as *Salmonella enterica* Typhimurium, *Pseudomonas aeruginosa*, *Bacillus cereus, E. coli*, *Staphylococcus epidermidis,* or *Vibrio cholera* [10]. Our team has formerly reported bacteriocin production in the case of L5 Kombucha isolate [19], tested against *Streptococcus thermophilus.* Such strains and their bacteriocin can be used in food and feed industries as natural biopreservatives and for probiotic application to humans or livestock, including functional foods. All Kombucha isolates proved to have high inhibitory activity against the major foodborne pathogens, like *Salmonella enterica* Typhimurium, *Listeria monocytogenes, Listeria ivanovii, Bacillus cereus, Proteus hauseri,* as well as on resistant or methicillin sensible *Staphylococcus aureus*. As a novelty, we have proven the inhibitory activity of the *P. pentosaceus* isolated from Kombucha on the human emerging pathogen, *Listeria ivanovii,* known as ruminants’ pathogen, as it can cause epidemic abortion, stillbirths, and encephalitis [35].

The LAB inhibitory activity on foodborne molds has often been reported on *Lactobacillus* spp. [36] and only occasionally in the case of *P. pentosacesus* or *P. acidilactici* isolated from natural sources. For instance, bacteriocin produced by *P. acidilactici* isolated from vacuum packed fermented meat products inhibited *A. fumigatus, A. parasiticus, F. oxyporum,* and *Penicillium* spp. [37], while *P. pentosaceus* isolated from malted cereals inhibited *Pencillium expansum* [38]. Strains from stored wheat samples showed antagonistic activity against different species such as *Alternaria alternata, Penicillium chrysogenum,* and *Aspergillus carbonarius* [39]. Although the same authors did not report any inhibition of *P. pentosacesus* on *A. niger,* our experiments found that the Kombucha LAB isolates exhibited low inhibitory activity, probably due to the low pH level of the suspension (pH of 4–4.5). There might be a correlation between our results and data reported on how Kombucha itself, made of green tea, and with a final pH of 3.5, have moderate inhibitory potential on molds like *Botrytis cinerea, Aspergillus carbonarius,* or *Penicillium expansum,* which proved on-plate and by in vivo artificial infections on grapes [40]. Both selected *Pediococcus* isolates (L3 and L5) have moderate to high inhibitory activity against *Penicillium expansum* and *Penicillium digitatum*, which suggest that our strains can be used in agricultural practices to control post-harvest mold development

The radical scavenging activity of LAB isolates is due to the colonization of viable cells and their propagation in the gut. Our results are comparable with studies reported before and Kombucha *Pediococcus* strains, such as L5, F2, and L3 LAB, which have an antioxidant activity of 56–58%. LAB isolated from *Neera* (fermented coconut palm nectar) can reach hydroxyl-scavenging activity of 32–77% [41], while for *Pediococcus* strains from *Omegisool* the DPPH radical-scavenging activity ranged between 30% and 39% [22].

Due to safety considerations, the obtained isolates were also tested for antibiotic resistance. The transmission of antibiotic resistance genes to potentially pathogenic bacteria in the gut is a major health concern related to the probiotic application [42]. The European Food Safety Authority (EFSA) recommends that bacterial strains harboring transferable antibiotic resistance genes should not be used in animal feeds or fermented and probiotic foods for human use. For an appropriate selection of functional strains, two main antibiotics’ groups are recommended in EFSA guidelines to be tested, such as inhibitors of protein synthesis (chloramphenicol, gentamycin, clindamycin, erythromycin, streptomycin, kanamycin, and tetracycline) and inhibitors of cell wall synthesis (ampicillin and vancomycin). In the case of LAB isolated from different natural sources, such as fermented coconut palm nectar, and belonging to other species (*Lactobacillus brevis, Enterococcus durans, Leuconostoc lactis, Enterococcus lactis, and Enterococcus faecium*), chloramphenicol, vancomycin, and streptomycin were effective inhibitors [41]. *Pediococcus* isolated from *Omegisool* are shown to be resistant to chloramphenicol [22]. Both Kombucha *Pediococcus* isolates (L3 and L5) are sensitive to ampicillin, penicillin, erythromycin, and lincomycin, while a broad range of other antibiotics are not effective inhibitors (fluoroquinolones, amoxicillin/clavulanic acid, cephalexin, cefuroxime, amikacin, streptomycin, kanamycin, sulphamethoxazole, vancomycin, and tetracycline). In this regard, before using these isolates in food or feed formulations per EFSA guidelines, virulence and antimicrobial resistance genes should be verified to prevent the horizontal gene transfer for antibiotic resistance.

The incorporation of probiotic bacteria to food products represents a major technological challenge because of the known sensitivity of these microorganisms to salt, spices, and other substances used in its formulation. In relation to the NaCl tolerance, the most halophilic LAB (*Enterococcus* ssp., *Lactobacillus* ssp.) were isolated from seafoods and fermented meats, being able to grew under a NaCl concentration of more than 22% [7]. Meanwhile *L. fermentum* and *L. plantarum* isolated from fermented plants grew under NaCl concentrations of less than 6% [43]. Strains of *P. pentosaceus* isolated from various traditional Thai fermented foods containing fish and pork were reported as tolerant up to 14% NaCl in an acid medium (pH 2) with 0.3–0.5% bile salt [10]. Our Kombucha isolates (L3 and L5) tolerated 5% NaCl. Furthermore, L5, after a period of adaptation, is tolerant to 7.5% NaCl. Thus, we concluded that L3 and L5 were resistant to salt concentrations used in industrial levels and maintained a concentration suitable for carrying probiotic effect.

According to Champagne et al. (2011) [44], a product containing probiotic organisms is efficient if it contains a number of viable cells higher than 10^6^–10^8^ CFU/g. However, viability and optimum concentration of probiotic microorganism is still under debate, but the trend is to have a minimum of one billion viable cells per 100 g of product to declare it as a probiotic functional product [45]. Lyophilization is one of the procedures used to deliver probiotics for commercial products. Regarding the lyophilization procedure, the survival rate may be improved by the use of other cryoprotectants, such as poly-glutamic acid, which was successfully used for the protection of probiotic *Lactobacillii* [45]. In the case of our *Pediococcus* isolates, by the use of glucose or sucrose as cryopretectant, we obtained a good viability rate of 86–92%. In addition, lyophilization did not affect the inhibitory activity on the tested pathogenic microorganisms. The initial biomass of 10^10^ CFU/mL conditioned by freeze-drying procedure, recovered levels of 10^9^ CFU/g after rehydration. Rehydration is a normal industrial step when using such dried strains, and has a major influence on the CFU readings obtained. We concluded that our strains were suitable to be conditioned by lyophilization and employed industrially after rehydration.

From a larger biotechnological point of view, probiotic bacteria can also be used as ingredients in cosmetic products. *P. acidilactici* strain isolated from Korean Perilla Leaf Kimchi was proven to a have direct melanin-degrading and tyrosinase-inhibiting effects, given that it has high value as a raw material for melanin degradation drugs and cosmetics [46]. However, there are still technical barriers to incorporate live probiotics into conventional skincare products with a reasonable shelf life. Some results are reported when using *Lactobacilli*, and the solution was to add probiotic ingredients which were not alive or viable to form colonies to the formulation at the end of the manufacturing process. Another strain of *P. pentosaceus* isolated from kaki fruit increased the antioxidative and aging activities of the *Lavandula angustifolia* extract through fermentation, so it was proposed to be used as an anti-aging agent [47]. Our *Pediococcus* isolates are expected to be further tested for such properties for further industrial application.

## 5. Conclusions

In our effort to find new natural resources and to develop new biotechnological solutions for food and pharmaceutical industries, we investigated several *Pediococcus* strains isolated from a local Kombucha source. Two of the strains, L3 (*P. penteosaceus*) and L5 (*P. acidilactici*), proved to have properties related to their potential use in the development of functional products, due to their tolerance to acid pH, the presence of pepsin and bile salts, resistance to a large range of different antibiotic classes, high antioxidant potential, and inhibitory activity among a large range of foodborne bacteria and fungi. Both strains were resistant to high NaCl concentrations and to the invasive lyophilization procedure, being good candidates for the industrial use.

## Figures and Tables

**Table 1:** Pathogenic microorganisms used in the antagonistic tests.

Strain	Origin	Special Characteristics
**Bacteria**		
*Escherichia coli* ATCC 8739	ATCC^®^	-
*Salmonella enterica* Typhimurium ATCC 14028	ATCC^®^	-
*Staphylococcus epidermidis* ATCC 12228	ATCC^®^	vancomycin sensitive
*Staphylococcus epidermidis* ATCC 51625	ATCC^®^	Methicillin resistant
*Staphylococcus aureus* ATCC 25923	ATCC^®^	methicillin sensitive
*Staphylococcus aureus* ATCC 6538	ATCC^®^	methicillin sensitive
*Staphylococcus aureus* ATCC 43300	ATCC^®^	methicillin and oxacilin -resistant
*Staphylococcus aureus* ATCC 33592	ATCC^®^	methicillin and gentamicin -resistant
*Listeria ivanovii* ATCC 19119	ATCC^®^	resistant in acid medium
*Listeria monocytogenes* ATCC 7644	ATCC^®^	serogroup 1/2c
*Proteus hauseri (vulgaris)* ATCC 13315	ATCC^®^	-
*Streptococcus pyogenes* ATCC 19615	ATCC^®^	β-hemolytic
*Bacillus cereus* CP1	UASVM Bucharest	-
**Yeast**		
*Candida albicans* ATCC 10231	ATCC^®^	serotype A
*Candida parapsilosis* ATCC 20019	ATCC^®^	-
*Candida guilermondii* MI 40	UASVM Bucharest	-
*Candida krusei* MI 41	UASVM Bucharest	-
**Molds**		
*Aspergillus niger* M4	UASVM Bucharest	
*Aspergillus carbonarius* MI 15	UASVM Bucharest	
*Aspergillus flavus* MI 24	UASVM Bucharest	
*Penicillium digitatum* MI 22	UASVM Bucharest	
*Penicillium expansum* MI BB Huși	UASVM Bucharest	

**Table 2:** Inhibitory activity of Kombucha LAB isolates on pathogenic microorganisms were tested using an adapted disk diffusion method.

Pathogenic Microorganisms	S1	S2	S3	L3	L5	F1	F2
**Bacteria**							
*Escherichia coli* ATCC 8739	+	+	+	+	+	+	+
*Salmonella enterica* Typhimurium ATCC 14028	**+ + +**	**+ ++**	**+ + +**	**+ + +**	**+ +**	**+ + +**	**+ + +**
*Staphylococcus epidermidis* ATCC 12228	+ +	+ +	+ +	+ +	+ +	+ +	+ +
*Staphylococcus epidermidis* ATCC 51625	+ +	+ +	+ +	+ +	+ +	+ +	+ +
*Staphylococcus aureus* ATCC 25923	**+ + +**	**+ + +**	**+ + +**	**+ + +**	**+ + +**	**+ + +**	**+ + +**
*Staphylococcus aureus* ATCC 6538	+ +	**+ + +**	**+ + +**	**+ + +**	**+ + +**	**+ + +**	+ +
*Staphylococcus aureus* ATCC 43300	**+ + +**	**+ + +**	**+ + +**	**+ + +**	**+ + +**	**+ + +**	**+ + +**
*Staphylococcus aureus* ATCC 33592	+ +	+ +	+ +	**+ + +**	**+ + +**	+ +	**+ + +**
*Listeria ivanovii* ATCC 19119	**+ + +**	**+ + +**	**+ + +**	**+ + +**	**+ + +**	**+ + +**	**+ + +**
*Listeria monocytogenes* ATCC 7644	**+ + +**	**+ + +**	**+ + +**	**+ + +**	**+ + +**	**+ + +**	**+ + +**
*Proteus hauseri (vulgaris)* ATCC 13315	**+ + +**	**+ + +**	**+ + +**	**+ + +**	**+ + +**	**+ + +**	**+ + +**
*Streptococcus pyogenes* ATCC 19615	**+ + +**	**+ + +**	**+ + +**	**+ + +**	**+ + +**	**+ + +**	**+ + +**
*Bacillus cereus* CP1	**+ + +**	**+ + +**	**+ + +**	**+ + +**	**+ + +**	**+ + +**	**+ + +**
**Yeast**							
*Candida albicans* ATCC 10231	+	++	+	++	++	+	+
*Candida parapsilosis* ATCC 20019	+	+	+	++	++	+	+
*Candida guilermondii* MI 40	+	+	+	++	++	+	+
*Candida krusei* MI 41	-	-	-	-	-	-	-
**Molds**							
*Aspergillus niger* M4	+	+	-	+	+	-	+
*Aspergillus carbonarius* MI 15	+	+	+	++	++	++	+
*Aspergillus flavus* MI 24	+	+	-	+	+	-	-
*Penicillium digitatum* MI 22	+++	++	++	+++	+++	+++	+++
*Penicillium expansum* MI BB Huși	+++	+++	++	++	+++	+++	+++

Legend: (-) = no halo formation; (+) inhibition halo of 1–5 mm diameter; (++) halo of 6–17 mm diameter; (+++) halo of 18–29 mm diameter.

**Table 3:** Antibiotic susceptibility of LAB strains of *Pediococcus* spp. isolated from industrial Kombucha.

Antibiotic Classes/Antibiotic	LAB Isolates	
	L3 *P. pentosaceus*	L5 *P.acidilactici*
**P** **enicillins**		
Ampicillin 10 µg/disc	MS	S
Penicillin 2 µg/disc	R	S
Amoxicillin/Clavulanic acid 20/10 µg/disc	R	R
**Cephalosporins**		
Cephalexin 30 µg/disc	R	R
Cefuroxime 30 µg/disc	R	R
Ceftriaxone 30 µg/disc	MS	MS
**Fluoroquinolones**		
Ciprofloxacin 1 µg/disc	R	R
Norfloxacin 30 µg/disc	R	R
Nalidixic acid 30 µg/disc	R	R
**Aminoglycosides**		
Amikacin 10 µg/disc	R	R
Gentamicin 10 µg/disc	MS	MS
Streptomycin 10 µg/disc	R	R
Kanamycin 30 µg/disc	R	R
**Macrolides**		
Erythromycin 10 µg/disc	S	S
**Lincosamide**		
Lincomycin 10 µg/disc	S	S
**Sulfonamides**		
Sulphamethoxazole 25 µg/disc	R	R
**Glycopeptides**		
Vancomycin 10 µg/disc	R	R
**Tetracyclines**		
Tetracycline 30 µg/disc	R	MS
Oxytetracycline 30 µg/disc	MS	MS
**Other**		
Chloramphenicol 30 µg/disc	R	MS
Colistin 10 µg/disc	R	R
Bacitracin 10 U	R	MS
Fluconazole 10 µg/disc	R	R
Nitrofurantoin 300 µg/disc	R	MS

Legend: R, resistant; S, sensitive; MS, moderately sensitive.

**Table 4:** Inhibitory activity of lyophilized Kombucha LAB isolates L3 and L5 on pathogenic microorganisms tested by adapted disk diffusion method.

Pathogenic Microorganisms	L3	L5
*Listeria ivanovii* ATCC 19119	**+ + +**	**+ + +**
*Listeria monocytogenes* ATCC 7644	**+ + +**	**+ + +**
*Salmonella enetrica* Typhimurium ATCC 14028	**+ + +**	+ +
*Staphylococcus aureus* ATCC 25923	**+ + +**	**+ + +**
*Staphylococcus aureus* ATCC 6538	**+ + +**	**+ + +**
*Staphylococcus aureus* ATCC 43300	**+ + +**	**+ + +**
*Staphylococcus aureus* ATCC 33592	**+ + +**	**+ + +**
*Bacillus cereus* CP1	**+ + +**	**+ + +**
*Candida albicans* ATCC 10231	++	++

Legend: (-) = no halo formation; (+) inhibition halo of 1–5 mm diameter; (++) halo of 6–17 mm diameter; (+++) halo of 18–29 mm diameter.

## Supplementary Materials

The following are available online at https://www.mdpi.com/2304-8158/9/12/1780/s1, Figure S1: On-plate aspects of the Kombucha LAB inhibitory activity on *Candida albicans* ATCC 10231 (Ca), *Staphylococcus aureus* ATCC 33592 methicilin resistant (Sa 33592), *Staphylococcus aureus* ATCC 25923 methicilin sensitive (Sa 25923), *Salmonella typhimurium* ATCC 14028 (St) tested by adapted disk diffusion assay, Figure S2: Aspects of the Kombucha LAB inhibitory activity on *Aspergillus carbonarius* MI 15 (left) and *Penicillium digitatum* MI 22 (right) tested by adapted disk diffusion assay, Figure S3: On-plate test of haemolytic activity of Kombucha LAB isolates on blood agar mediu, Table S1: Results of the molecular identification of Kombucha LAB F1 and F2 by 16S rDNA sequencing with Lac primers.
