# Supplementary material for: The Biotechnological Potential of Pediococcus spp. Isolated from Kombucha Microbial Consortium
Source: Foods. 2020 Dec 1;9(12):1780. doi: 10.3390/foods9121780 (PMC7760545; doi:10.3390/foods9121780)
Supplement: Supplementary file 1 [file foods-09-01780-s001.pdf]

Supplementary data

The biotechnological potential of *Pediococcus* ssp. isolated from Kombucha microbial consortium

Camelia Filofteia Diguță[1], George Daniel Nițoi[1], Florentina Matei[1]*, Gabriela Luță[1] and Călina Petruța Cornea[1]

Faculty of Biotechnologies, University of Agronomic Sciences and Veterinary Medicine of Bucharest, 011464, Romania; camifilo@yahoo.com (C.F.D); daniel.nitoi0@gmail.com (G.D.N); florentina.matei@biotehnologii.usamv.ro (F.M); gluta@yahoo.com (G.L); pccornea@yahoo.com (C.P.C)
* Correspondence: florentina.matei@biotehnologii.usamv.ro

2. Materials and Methods

2.1. Microorganisms

Sequencing Results for F1 and F2

Table S1. Results of the molecular identification of Kombucha LAB F1 and F2 by 16S rDNA sequencing with Lac primers (according to Matei et al. 2018 [19])

Sequences ID	% Sequence Alignment (https://www.ebi.ac.uk/Tools/msa/clustalo/)	Identity (%) (https://blast.ncbi.nlm.nih.gov/Blast.cgi)
F1_LacF	95.82	*Pediococcus pentosaceus* (100%)
F1_LacR		*Pediococcus pentosaceus* (99.29%)
F2_LacF	95.77	*Pediococcus acidilactici* (99.32%)
F2_LacR		*Pediococcus acidilactici* (100%)

3.3. Inhibitory activity of Kombucha LAB on pathogenic microorganisms

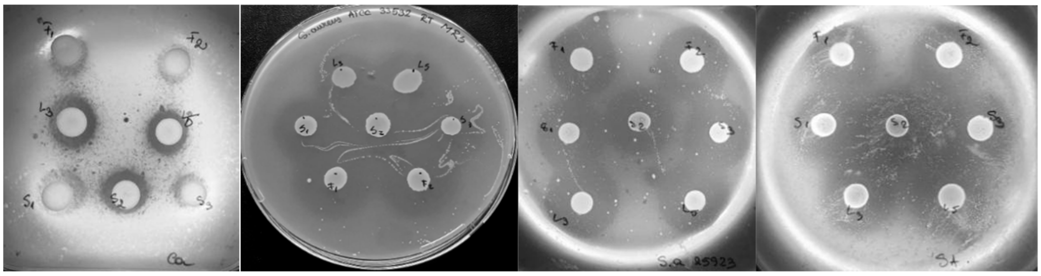

Figure S1. On-plate aspects of the Kombucha LAB inhibitory activity on *Candida albicans* ATCC 10231 (Ca), *Staphylococcus aureus* ATCC 33592 methicilin resistant (Sa 33592), *Staphylococcus aureus* ATCC 25923 methicilin sensitive (Sa 25923), *Salmonella typhimurium* ATCC 14028 (St) tested by adapted disk diffusion assay.

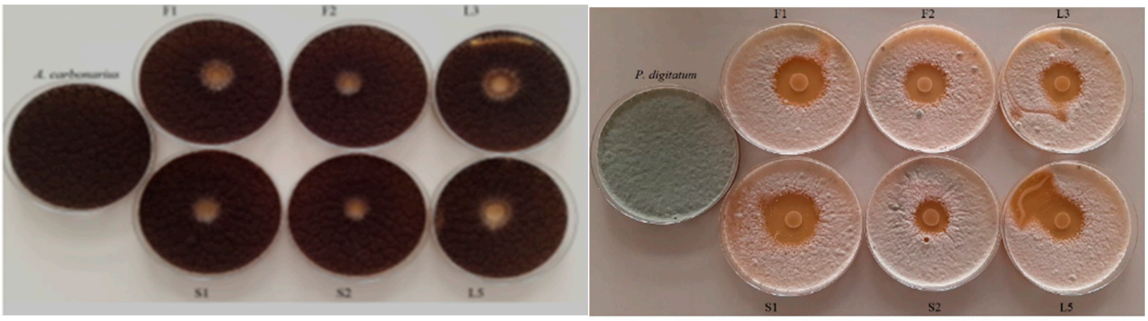

Figure S2. Aspects of the Kombucha LAB inhibitory activity on *Aspergillus carbonarius* MI 15 (left) and *Penicillium digitatum* MI 22 (right) tested by adapted disk diffusion assay.

3.4. Hemolytic and antioxidant activity of Kombucha LAB Strains

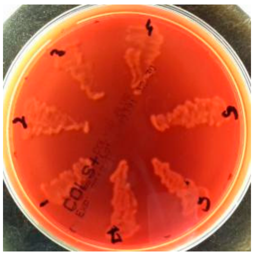

Figure S3. On-plate test of haemolytic activity of Kombucha LAB isolates on blood agar mediu (1- S1; 2- S2; 3- S3; 4-L3; 5-L5; 6-F1; 7-F2).
